# Valor Prognóstico da Escore PRECİSE DAPT em Resultados de Curto e Longo Prazo em Pacientes MINOCA com Síndrome Coronariana Aguda

**DOI:** 10.36660/abc.20230791

**Published:** 2024-05-07

**Authors:** Tolga Onuk, Fuat Polat, Bariş Yaylak, Ali Nazmi Çalik, Semih Eren, Şükrü Akyüz

**Affiliations:** 1 Dr. Siyami Ersek Thoracic and Cardiovascular Surgery Education Research Hospital Istanbul Turquia Dr. Siyami Ersek Thoracic and Cardiovascular Surgery Education Research Hospital, Istanbul – Turquia; 2 Okan University Faculty of Health Science Istanbul Turquia Okan University Faculty of Health Science, Istanbul – Turquia

**Keywords:** MINOCA, Fibrilação Atrial, Infarto do Miocárdio, Terapia Antiplaquetária Dupla

## Abstract

**Fundamento:**

O infarto do miocárdio com artérias coronárias não obstrutivas (MINOCA) constitui um subconjunto significativo de infartos agudos do miocárdio (IAM) com marcadores prognósticos incertos. A avaliação precoce do risco é crucial para identificar pacientes MINOCA em risco de resultados adversos.

**Objetivos:**

Este estudo teve como objetivo avaliar a capacidade preditiva do escore PRECISE-DAPT na avaliação do prognóstico de curto e longo prazo em pacientes MINOCA que apresentam infarto do miocárdio sem supradesnivelamento do segmento ST (IAMSSST) ou com supradesnivelamento do segmento ST (IAMCSST).

**Métodos:**

Entre 741 pacientes MINOCA, o escore PRECISE-DAPT foi calculado para analisar sua associação com eventos cardiovasculares adversos maiores (MACE) intra-hospitalares e de acompanhamento. Os parâmetros que apresentaram significância nos grupos MACEM (+) foram submetidos à análise estatística: regressão logística univariada para eventos intra-hospitalares e regressão univariada de Cox para eventos de seguimento. Para significância estatística, foi adotado nível pré-definido de α = 0,05. Os parâmetros que demonstraram significância foram submetidos à regressão logística múltipla para eventos intra-hospitalares e à regressão multivariada de Cox para eventos de seguimento.

**Resultados:**

Os MACE intra-hospitalares ocorreram em 4,1% dos pacientes, enquanto 58% apresentaram MACE no acompanhamento. Os níveis de hemoglobina e o escore PRECISE-DAPT foram identificados como parâmetros independentes para MACE intra-hospitalar. Além disso, a fração de ejeção (FE%) e o escore PRECISE-DAPT surgiram como preditores independentes de MACE no acompanhamento.

**Conclusões:**

O estudo revelou que um escore PRECISE-DAPT mais alto foi significativamente associada a riscos aumentados de eventos cardiovasculares adversos maiores tanto intra-hospitalares quanto de longo prazo em pacientes MINOCA que apresentam síndrome coronariana aguda (SCA), ressaltando o potencial do escore na estratificação de risco para esta coorte de pacientes.

**Figure:**
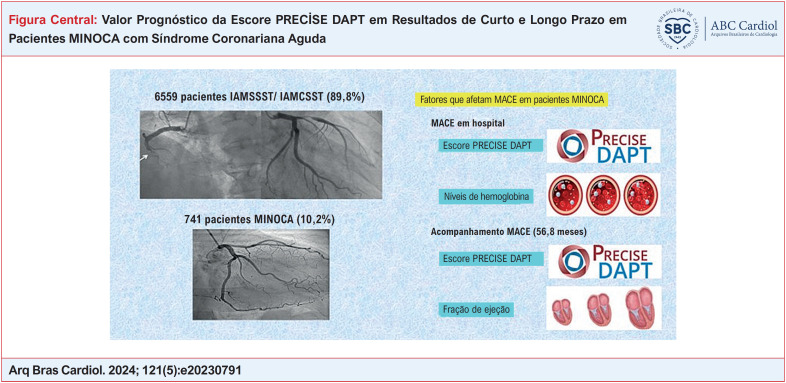


## Introdução

Pacientes com infarto do miocárdio com artérias coronárias não obstrutivas (MINOCA) representam 6% a 14% de todos aqueles com infarto agudo do miocárdio (IAM).^1 - 4^

O diagnóstico de MINOCA depende da presença de IAM clínico e da ausência de doença arterial coronariana obstrutiva (DAC). Na verdade, MINOCA é inicialmente considerado um diagnóstico válido durante a angiografia até que outras possíveis causas de elevação da troponina sejam excluídas. As causas subjacentes podem ser condições coronarianas ou não coronarianas, incluindo doenças cardíacas ou não cardíacas.^5^ MINOCA pode ser apresentado como infarto do miocárdio com supradesnivelamento do segmento ST (IAMCSST) ou sem supradesnivelamento do segmento ST (IAMSSST) em um eletrocardiograma (ECG), entretanto, IAMCSST é mais comum na população MINOCA.^4 , 6 , 7^ Pacientes MINOCA apresentam melhor prognóstico em comparação aos pacientes com síndrome coronariana aguda (SCA) com DAC obstrutiva.^8 - 11^ Entretanto, pacientes MINOCA apresentam pior expectativa de vida em comparação com indivíduos saudáveis de mesma idade e sexo.^8 - 11^

Foi demonstrado anteriormente que escores de risco como o Grace Risk Score estão associados ao prognóstico na população MINOCA.^12^ O escore PRECISE-DAPT, que prevê risco de sangramento em pacientes submetidos a implante de Stent e subsequente terapia antiplaquetária dupla (DAPT), foi desenvolvido para prever o risco de sangramento em pacientes tratados com DAPT após intervenção coronária percutânea (ICP). Esse escore é calculado por cinco itens (idade, contagem de leucócitos, nível de hemoglobina, depuração de creatinina e história de sangramento espontâneo) e pacientes com escore > 25 apresentam alto risco de sangramento.^13^ As diretrizes atuais recomendam o uso do escore PRECISE-DAPT para estratificação de risco de sangramento, e um escore > 25 indica que o período DAPT deve ser menor que 3 a 6 meses em pacientes com escore < 25,^14 - 16^ Em um estudo recente, um escore PRECISE-DAPT alto foi associado a maior mortalidade por todas as causas em longo prazo em pacientes com infarto agudo do miocárdio (IAM).^17^ Assim, o presente estudo tem como objetivo avaliar o desempenho do escore PRECISE-DAPT na predição do prognóstico de curto e longo prazo em pacientes MINOCA apresentando SCA.

## Métodos

**Figura 1:**
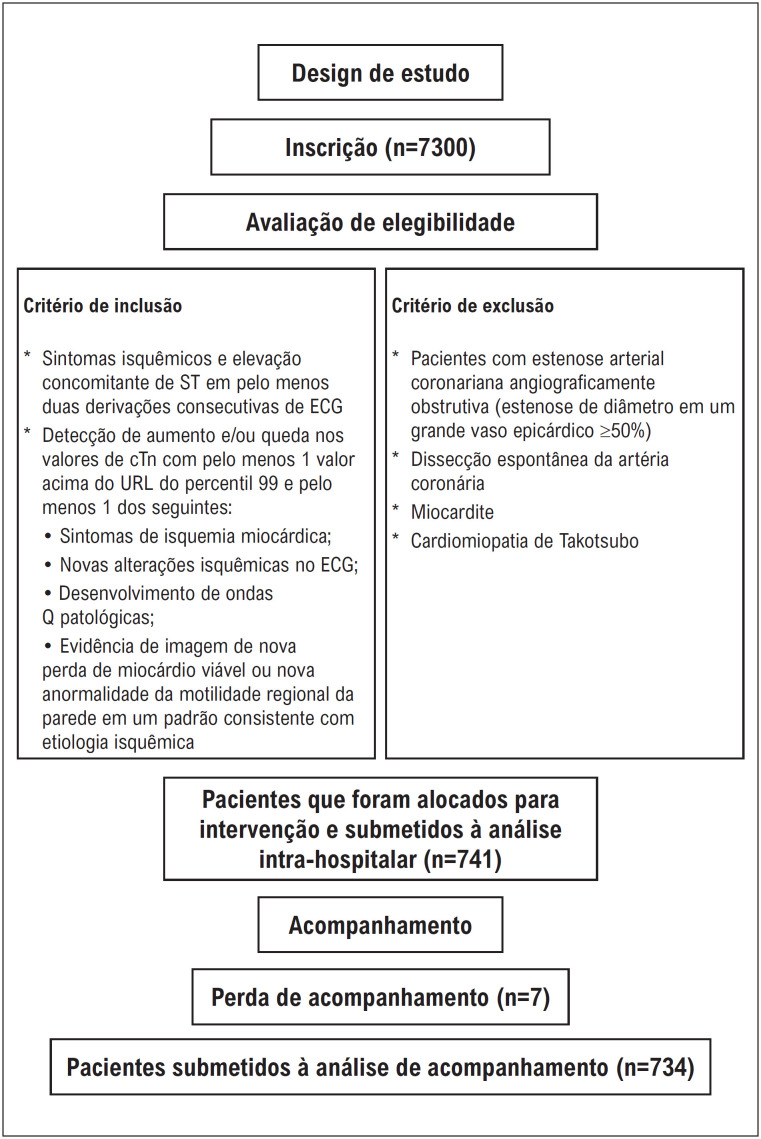
Fluxograma da população do estudo.

O estudo é retrospectivo e observacional. Incluímos 7.300 pacientes internados em nosso hospital com diagnóstico de IAMCSST OU ISMSSST entre abril de 2013 e dezembro de 2022. Entre os 7.300 pacientes submetidos à intervenção percutânea, um subconjunto de 741 indivíduos recebeu diagnóstico de SCA e MINOCA. A angiografia coronária foi realizada em todos os pacientes. Pacientes que não apresentavam estenose coronariana de 50% ou mais em qualquer artéria coronária na angiografia coronariana e que não foram diagnosticados com dissecção espontânea da artéria coronária, miocardite e cardiomiopatia de Takotsubo foram classificados como MINOCA. Em nosso estudo, pacientes com estenose obstrutiva da artéria coronária, exceto doenças ateroscleróticas da artéria coronária, não foram incluídos no estudo e não foram classificados como MINOCA. O fluxograma da população do estudo é apresentado na Figura 1 .

O escore PRECISE-DAPT, idade, depuração de creatinina, contagem de leucócitos, hemoglobina e histórico de sangramento prévio foram calculados para cada caso usando uma calculadora da web ( http://www.precisedaptscore.com ). Dados clínicos, bioquímicos, intervencionistas, ECG e ecocardiografia transtorácica foram obtidos do conjunto eletrônico de dados do hospital. A ecocardiografia foi realizada para determinar a fração de ejeção do ventrículo esquerdo (FEVE). A proporção de pacientes com história prévia de ataque cardíaco ou revascularização coronariana foi especificada e incluída no estudo. Todos os pacientes, inclusive aqueles com infarto prévio ou histórico de revascularização, foram submetidos a ICP de emergência. Os medicamentos, incluindo o início e a duração da terapia antiplaquetária dupla e da terapia anticoagulante, foram prescritos a critério do médico assistente no momento da alta. Os principais eventos cardiovasculares intra-hospitalares foram identificados como mortalidade cardiovascular, acidente vascular cerebral e sangramento maior. Para avaliação de eventos cardiovasculares maiores de longo prazo foram considerados: morte vascular cardíaca, morte não cardiovascular, acidente vascular cerebral, sangramento maior, intervenção percutânea e infarto do miocárdio. O MACE intra-hospitalar de todos os pacientes foi obtido dos prontuários médicos do paciente. Os principais eventos cardiovasculares de longo prazo de todos os pacientes foram obtidos dos registros médicos do paciente, dos médicos relevantes do hospital para o qual os pacientes foram encaminhados ou por telefone dos pacientes. O desenho do estudo foi aprovado pelo comitê de ética local e conduzido de acordo com a Declaração de Helsinque.

### Análise estatística

A análise dos dados utilizou o software SPSS (versão 23.0, SPSS, Inc., Chicago, IL). As variáveis contínuas foram avaliadas quanto à normalidade, empregando inspeção visual por meio de histogramas, gráficos de probabilidade e teste de Kolmogorov-Smirnov. O teste de Levene foi utilizado para avaliar a homogeneidade. As variáveis contínuas foram apresentadas como média ± desvio padrão para dados com distribuição normal e como mediana com intervalo interquartil para dados com distribuição não normal.

Além disso, testes estatísticos incluindo o teste t de Student não pareado e o teste de Mann-Whitney foram aplicados para avaliar diferenças nas variáveis contínuas entre os grupos, garantindo uma análise abrangente das associações de variáveis contínuas. Enquanto as variáveis categóricas foram dadas como frequências absolutas e relativas, as diferenças nas variáveis categóricas entre os grupos foram avaliadas pelo teste qui-quadrado ou exato de Fisher.

Na análise intra-hospitalar, os parâmetros que demonstraram diferença estatisticamente significativa no grupo MACE (+) foram submetidos à avaliação inicial por meio de análise de regressão logística univariada, seguida de análise de regressão logística múltipla para resultados significativos. Para a análise de acompanhamento, os parâmetros que exibiram uma diferença estatisticamente significativa no grupo MACE (+) foram inicialmente examinados usando análise de regressão univariada de Cox, seguida por regressão multivariada de Cox para resultados significativos.

Posteriormente, os fatores que predizem independentemente o MACE foram submetidos à análise ROC para determinar a sensibilidade e a especificidade. O valor de corte do escore PRECISE-DAPT, determinado pela maior sensibilidade e especificidade, foi utilizado na análise de Kaplan-Meier para comparar a sobrevida em longo prazo. O nível de significância adotado na análise estatística foi de 5%. Além disso, foram realizadas análises garantindo pressupostos básicos para regressão logística, incluindo independência de erros, linearidade no logit para variáveis contínuas, ausência de multicolinearidade e ausência de outliers fortemente influentes.

## Resultado

**Figura 2:**
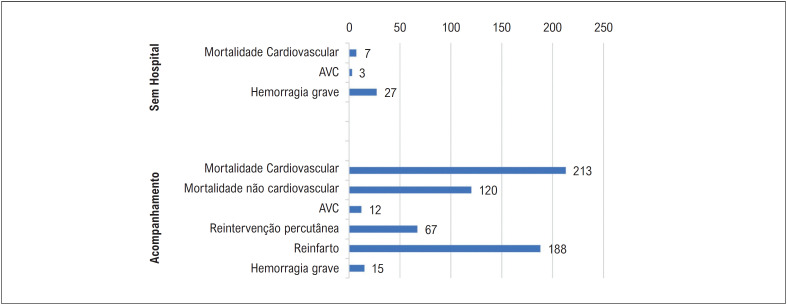
MACE intra-hospitalar e de acompanhamento dos pacientes. AVC: acidente vascular cerebral.

Um total de 741 pacientes foram incluídos no estudo. Foram obtidos dados de acompanhamento de longo prazo de 734 pacientes. A mediana do período de acompanhamento do total de 734 pacientes incluídos no estudo foi de 56,8 (intervalo interquartil [IQR] 38,3-76,5) meses. Os MACE intra-hospitalares ocorreram em 4,1% dos pacientes e os MACE no acompanhamento ocorreram em 58% dos pacientes ( Figura 2 ).

A idade média dos pacientes foi de 61,4±14,1 anos, e o sexo masculino foi predominante (58,5%). A média do escore PRECISE DAPT dos pacientes foi de 23,6 ± 14,4. Quase todos os pacientes (98,8%) receberam AAS 300 mg antes ou durante o procedimento. A maioria dos pacientes recebeu Clopidogrel 300 mg antes ou durante o procedimento. A proporção de pacientes que receberam Ticagrelor no início do estudo foi de 11,7%. Outras características de todos os pacientes são mostradas na Tabela 1 .

**Tabela 1:** Características clínicas e demográficas dos pacientes

Características do paciente	n = 741
Sexo (masculino) (%)	434(58,5)
Idade (anos) (média±DP)	61,4±14,1
IMC (kg/m²) (média±DP)	28,21±5,35
Hipertensão (%)	369(49,7)
Diabetes Mellitus (%)	224(30,2)
Tabagismo (Ativo) (%)	305(41,1)
Dislipidemia (%)	39(5.3)
DPOC (%)	29(3,9)
Choque cardiogênico na admissão (%)	40(5,4)
Killip III-IV na admissão (%)	42(5,7)
BRE no ECG (%)	68(9,2)
Ataque cardíaco prévio (%)	49(6,6)
Revascularização prévia (%)	71(9,6)
Acidente vascular cerebral prévio (%)	29(3,9)
Escore PRECISE DAPT (média±DP)	23,6±14,4
Uso prévio de varfarina (%)	42(5,7)
Uso prévio de NOAC (%)	29(3,9)
Leucócitos (10^3^/uL) (média±DP)	10,3±4,1
Creatinina (mg/dL) (mediana, IQR)	1,0(0,8-1,3)
Hemoglobina (g/dL) (média±DP)	12,5±2,1
FEVE% (média±DP)	46±12,7
Ticagrelor (%)	87(11,7)
Clopidogrel 300 mg (%)	638(86)
Clopidogrel 600 mg (%)	8(1.1)
AAS (%)	733(98,8)
Betabloqueador (%)	693(93,4)
Bloqueador dos canais de cálcio (%)	172(23,2)
IECA/BRA (%)	574(77,4)
IBP (%)	734(99,1)
Estatina (%)	618(83,3)
HBPM antes do procedimento (%)	667(90,6)

IECA/BRA: Inibidores da enzima conversora de angiotensina/bloqueadores dos receptores da angiotensina II; AAS: Ácido acetilsalicílico; IMC: Índice de massa corporal; DPOC: Doença pulmonar obstrutiva crônica; ECG: Eletrocardiografia; IQR: Intervalo interquartil; BRE: Bloqueio de ramo esquerdo; HBPM: Heparina de baixo peso molecular; FEVE: fração de ejeção do ventrículo esquerdo; NOAC: anticoagulantes orais não antagonistas da vitamina K; IBP: inibidor da bomba de prótons; DP: desvio padrão.

Os pacientes foram divididos em dois grupos com (+) MACE hospitalar e sem (-) MACE hospitalar ( Tabela 2 ). No grupo MACE (+) havia taxas mais altas de choque cardiogênico (p=0,008), maior média de IMC (p=0,025) e fumantes ativos. A escore PRECISE DAPT foi duas vezes maior (22,6% vs. 43,9%) em pacientes MACE (+) do que em pacientes MACE (-). Além disso, os pacientes com MACE (+) apresentaram taxas mais baixas de inibidores da ECA/BRA e prescrições de estatinas, níveis mais baixos de hemoglobina, FEVE% mais baixos e níveis mais elevados de creatinina.

**Tabela 2:** Características clínicas e demográficas dos pacientes com ou sem grupos de MACE intra-hospitalares

Características do paciente	MACE (-) no Hospital (n=709)	MACE (+) no Hospital (n=32)	Valor p
Sexo (masculino) (%)	414(58,4)	19(59,4)	0,533
Idade (anos) (média±DP)	61,6±14	57.1±14.3	0,071
IMC (kg/m²) (média±DP)	28.09±5.02	30.05±6,86	0,008 *
Hipertensão (%)	352(49,6)	17(53,1)	0,419
Diabetes Mellitus (%)	215(30,3)	9(28,1)	0,482
Tabagismo (Ativo) (%)	285(40,2)	19(59,4)	0,025 *
Dislipidemia (%)	38(5.4)	1(3.2)	0,488
DPOC (%)	28(3,9)	1(3.1)	0,640
Choque cardiogênico na admissão (%)	35(4,9)	5(15,6)	0,024 *
Killip III-IV na admissão (%)	37(5.2)	4(12,5)	0,094
BRE no ECG (%)	64(9)	4(12,5)	0,337
Ataque cardíaco prévio (%)	46(6,5)	3(9,4)	0,127
Revascularização prévia (%)	66 (9,3)	5(15,6)	0,089
Acidente vascular cerebral prévio (%)	28(3,9)	1(3.2)	0,640
Escore PRECISE DAPT (média±DP)	22.6±13,8	43,9±13,9	<0,001 *
Uso prévio de varfarina (%)	41(5,8)	1(3.1)	0,444
Uso prévio de NOAC (%)	28(4)	1(3.1)	0,640
Leucócitos (10^3^/uL) (média±DP)	10.2±4.1	11,5±4.1	0,053
Creatinina (mg/dL) (mediana, IQR)	0,9 (0,7-1,2)	1,3(0,9-1,6)	0,003 *
Hemoglobina (g/dL) (média±DP)	12.6±1,9	9.4±1.7	<0,001 *
FEVE% (média±DP)	46,3±12.4	38,2±15,5	<0,001 *
Ticagrelor (%)	83(11,7)	4(12,5)	0,531
Clopidogrel 300 mg (%)	609(85,9)	28(87,5)	0,525
Clopidogrel 600 mg (%)	8(1.1)	0(0)	0,701
AAS (%)	701(98,9)	31(96,99	0,329
Betabloqueador (%)	665(93,8)	28(87,5)	0,146
Bloqueador dos canais de cálcio (%)	161(22,7)	11(34,4)	0,098
IECA/BRA (%)	562(79,3)	12(37,5)	0,001 *
IBP (%)	702(99)	32(100)	0,733
Estatina (%)	597(84,2)	21(65,6)	0,011 *
HBPM antes do procedimento (%)	638(90,6)	29(90,6)	0,593

IECA/BRA: Inibidores da enzima conversora de angiotensina/bloqueadores dos receptores da angiotensina II; AAS: Ácido acetilsalicílico; IMC: Índice de massa corporal; DPOC: Doença pulmonar obstrutiva crônica; ECG: Eletrocardiografia; IQR: Intervalo interquartil; BRE: Bloqueio de ramo esquerdo; HBPM : Heparina de baixo peso molecular; FEVE: Fração de ejeção do ventrículo esquerdo; MACE: Principais eventos cardiovasculares adversos; NOAC: Anticoagulantes orais não antagonistas da vitamina K; IBP: Inibidor da bomba de prótons; DP: Desvio padrão.

* Valor P significativo.

Quando os parâmetros estatisticamente significativos entre os dois grupos foram avaliados por análise de regressão logística univariada, tabagismo ativo, choque na admissão, escore PRECISE DAPT, nível de creatinina, nível de hemoglobina, FEVE%, uso de IECA/BRA, uso de estatinas e IMC foram os fatores retidos significativamente. Aplicando esses parâmetros a análises de regressão logística múltipla, o nível de hemoglobina e o escore PRECISE DAPT foram considerados parâmetros independentes para MACE intra-hospitalar ( Tabela 3 ).

**Tabela 3:** Resultados da análise de regressão logística univariada e múltipla dos grupos com e sem MACE durante o período intra-hospitalar

Características do paciente	Análise Univariada			Análise de Regressão Logística Múltipla		
	OR	IC 95%	p	OR	IC 95%	p
Fumante (Ativo)	2.174	1.051-4.473	0,035			
Choque na admissão	3.566	1.299-9.820	0,014			
Escore PRECISA DAPT	1.100	1.071-1.131	<0,001	1.039	1.003-1.077	0,034
Creatinina	1.304	1.075-1.581	0,007			
Hemoglobina	0,405	0,316-0,520	<0,001	0,482	0,356-0,652	<0,001
FEVE	0,955	0,930-0,981	0,001			
IECA/BRA	0,157	0,075-0,328	<0,001			
Estatina	0,358	0,168-0,763	0,008			
IMC	1.073	0,030-5,522	0,019			

IECA/BRA: Inibidores da enzima conversora de angiotensina/bloqueadores dos receptores da angiotensina II; IMC: Índice de massa corporal; FEVE: Fração de ejeção do ventrículo esquerdo.

Os pacientes acompanhados foram divididos em dois grupos com (+) MACE de acompanhamento e sem (-) MACE de acompanhamento ( Tabela 4 ). Os MACE foram mais frequentes em pacientes com choque cardiogênico, Killip III-IV na apresentação e em pacientes com BRE no ECG. A escore PRECISE DAPT foi mais frequente (18,1% vs. 27,1%) em pacientes MACE (+) do que em pacientes MACE (-). Pacientes com MACE (+) apresentaram taxas mais altas de uso de varfarina e BCC e taxas mais baixas de uso de clopidogrel e ticagrelor por mais de 1 ano, uso de IECA/BRA e IBP. Além disso, foram observados níveis mais baixos de hemoglobina e FEVE% e níveis mais elevados de creatinina e leucócitos em pacientes com MACE (+).

**Tabela 4:** Características clínicas e demográficas dos pacientes com ou sem acompanhamento dos grupos MACE

Características do paciente	MACE (-) Acompanhamento (n=308)	MACE (+) Acompanhamento (n=426)	Valor p
Sexo (masculino) (%)	172(55,8)	258(60,6)	0,114
Idade (anos) (média±DP)	61,7±13.2	61,1±14,5	0,563
IMC (kg/m²) (média±DP)	28,7±5.2	27,8±4.9	0,113
Hipertensão (%)	156(50,6)	208(48,8)	0,340
Diabetes Mellitus (%)	93(30,2)	131(30,8)	0,469
Tabagismo (Ativo) (%)	123(39,9)	178(41,8)	0,335
Dislipidemia (%)	20(6,5)	19(4,5)	0,248
DPOC (%)	12(3,9)	17(4,0)	0,554
Choque cardiogênico na admissão (%)	9(2,9)	28(6,6)	0,018 *
Killip III-IV na admissão (%)	6(1,9)	32(7,5)	<0,001 *
BRE no ECG (%)	15(4,9)	52(12,2)	<0,001 *
Ataque cardíaco prévio (%)	20(6,5)	29(6,8)	0,405
Revascularização prévia (%)	29(9,4)	42(9,9)	0,234
Acidente vascular cerebral prévio (%)	12(3,9)	17(4,0)	0,554
Escore PRECISE DAPT (média±DP)	18,1±13,2	27,1±13,6	<0,001 *
Uso prévio de varfarina (%)	11(3,6)	31(7,3)	0,023 *
Uso prévio de NOAC (%)	11(3,6)	18(4.2)	0,406
Leucócitos (10^3^/uL) (média±DP)	9,5±3,8	10,4±4,3	0,008 *
Creatinina (mg/dL) (mediana, IQR)	1,0(0,8-1,3)	1,3(1,0-1,6)	<0,001 *
Hemoglobina (g/dL) (média±DP)	12,9±2,1	12,1±2,1	<0,001 *
FEVE% (média±DP)	51,5±10,6	42,2±12,4	0,001 *
AAS (%)	305(99)	420(98,6)	0,433
Betabloqueador (%)	291(94,5)	397(93,2)	0,291
Bloqueador dos canais de cálcio (%)	52(16,9)	120(28,2)	<0,001 *
IECA/BRA (%)	268(87)	306(71,9)	<0,001 *
IBP (%)	283(91,9)	333(78,2)	<0,001 *
Inibidor P2Y12 >1 ano (%)	214(69,5)	217(50,9)	<0,001 *

IECA/BRA: Inibidores da enzima conversora de angiotensina/bloqueadores dos receptores da angiotensina II; AAS: Ácido acetilsalicílico; IMC: Índice de massa corporal; DPOC: Doença pulmonar obstrutiva crônica; ECG: Eletrocardiografia; IQR: Intervalo interquartil; BRE: Bloqueio de ramo esquerdo; HBPM: Heparina de baixo peso molecular; FEVE: Fração de ejeção do ventrículo esquerdo; MACE: Principais eventos cardiovasculares adversos; NOAC: Anticoagulantes orais não antagonistas da vitamina K; IBP: Inibidor da bomba de prótons; DP: Desvio padrão.

* Valor p significativo.

Quando parâmetros estatisticamente significativos entre os dois grupos foram avaliados por análise de regressão univariada de Cox, BRE no ECG, escore PRECISE DAPT, nível de leucócitos, nível de creatinina, nível de hemoglobina, FEVE, uso de clopidogrel e ticagrelor por mais de 1 ano, uso de IECA/BRA e uso de estatinas foram os fatores retidos significativamente. Aplicando esses parâmetros a uma análise de regressão multivariada de Cox (método enter), descobriu-se que a FEVE% e o escore PRECISE DAPT são os preditores independentes de MACE de acompanhamento ( Tabela 5 ).

**Tabela 5:** Resultados da análise de regressão de Cox univariada e multivariada dos grupos com e sem MACE durante o acompanhamento

Características do paciente	Análise Univariada			Análise multivariada		
	HR	IC 95%	Valor p	HR	IC 95%	Valor p
Choque na admissão	1,383	0,942-2,030	0,097			
Killip III-IV na admissão	1,432	0,998-2,054	0,051			
BRE no ECG	1,381	1,024-1,862	0,034 *			
Escore PRECISA DAPT	1,019	1,013-1,025	<0,001 *	1,013	1,006-1,020	<0,001
Uso prévio de varfarina	1,096	0,760-1,581	0,624			
Leucócitos	1,027	1,006-1,048	0,012 *			
Creatinina	1,104	1,025-1,190	0,009 *			
Hemoglobina	0,902	0,862-0,945	0,001 *			
FEVE	0,975	0,968-0,982	<0,001 *	0,980	0,972-0,987	<0,001
CCB	1.319	1.067-1.630	0,010 *			
IECA/BRA	0,710	0,574-0,878	0,002 *			
Estatina	0,698	0,554-0,879	0,002 *			
Inibidor P2Y12 >1 ano	0,799	0,660-0,968	0,022 *			

IECA/BRA: inibidores da enzima conversora de angiotensina/bloqueadores dos receptores da angiotensina II; ECG: eletrocardiografia; BRE: bloqueio de ramo esquerdo; FEVE: fração de ejeção do ventrículo esquerdo.

* Valor p significativo.

**Figura 3:**
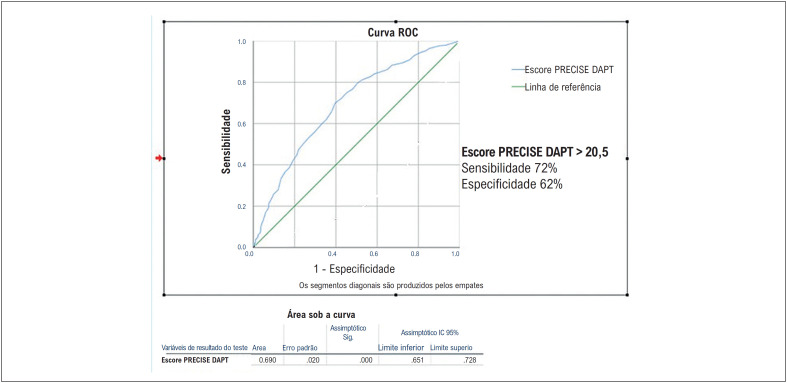
Análise ROC do escore PRECISE DAPT entre grupos na análise de regressão proporcional multivariada de Cox.

Avaliamos a sensibilidade e a especificidade dos preditores independentes de MACE por meio da análise ROC ( Figura 3 ). Como a área AUC foi baixa na análise ROC realizada de acordo com a FEVE, não foram feitos cálculos de sensibilidade e especificidade. O nível AUC da escore PRECISE DAPT foi suficientemente elevado. Quando o escore PRECISE DAPT foi > 20,5, a sensibilidade foi determinada como 72%, a especificidade como 62%, o valor preditivo positivo como 65,5% e o valor preditivo negativo como 68,9%.

**Figura 4:**
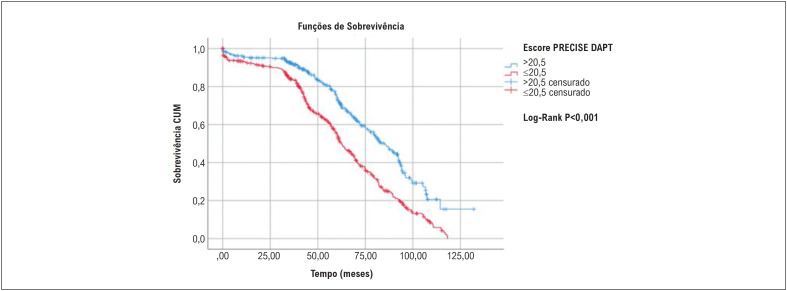
Sobrevida em longo prazo de acordo com o escore PRECISE DAPT pela análise de Kaplan-Meier.

Utilizamos o valor de corte de 20,5% para avaliar a sobrevida em longo prazo pela análise de Kaplan-Meier ( Figura 4 ), que resultou em um qui-quadrado de 43,29, p<0,001 de longo prazo. Observou-se que a taxa de MACE entre os grupos diferiu no primeiro ano de acompanhamento.

## Discussão

Neste estudo, descobrimos que um escore PRECISE-DAPT alto é um preditor independente significativo de MACE intra-hospitalar e de longo prazo em pacientes MINOCA com SCA. Até onde sabemos, este é o primeiro estudo a relatar que o MACE intra-hospitalar e de longo prazo é significativamente maior em pacientes MINOCA com SCA com um escore PRECISE-DAPT alto. A análise ROC demonstrou que o escore PRECISE-DAPT teve uma capacidade de discriminação moderada para estratificar pacientes com SCA-MINOCA pelo risco de MACE. Os dados mostraram que o escore de risco PRECISE-DAPT tem valor prognóstico em pacientes com SCA-MINOCA ( Figura Central ).

Numerosos estudos foram publicados recentemente sobre o valor prognóstico do MINOCA. Em uma revisão recente realizada por Pasupathy et al., o MINOCA apresentou uma taxa de mortalidade por todas as causas mais baixa do que aqueles com IAM-DAC.^4^ Em uma meta-análise publicada por Pizzi et al., todas as taxas de eventos de desfecho cardiovascular (MACE, todas as mortes, morte cardíaca, IAM e todas as mortes mais IAM) na DAC não obstrutiva foram significativamente menores do que aquelas na DAC obstrutiva.^18^ Um estudo recente em pacientes chineses do MINOCA revelou que MACE (mortes cardiovasculares, IAM não fatal, acidentes vasculares cerebrais, insuficiência cardíaca, e reinternações relacionadas a doenças cardiovasculares) foi menor no grupo MINOCA do que no grupo IAM-DAC no acompanhamento de 1 ano.^19^ Em alguns estudos, foi demonstrado que o prognóstico do MINOCA é melhor do que o dos pacientes IAM-DAC.^4 , 7 , 20^ Alguns estudos indicaram que pacientes com MINOCA tiveram resultados clínicos semelhantes aos de pacientes com IAM-DAC.^21^ Dreyer et al., indicaram pior prognóstico no acompanhamento de 1 ano após IAM em pacientes idosos com MINOCA com mais de 65 anos de idade em comparação com aqueles com pacientes com IAM-DAC.^22^ Além de todos esses estudos, 2 estudos encontraram maior incidência de MACE em pacientes MINOCA em comparação com a população normal.^23 , 24^

As ferramentas de avaliação de risco podem ajudar a prever a probabilidade de mortalidade e morbidade. Até onde sabemos, existe atualmente apenas um escore de risco para prever o prognóstico do MINOCA. Yin, Guoqing, et al., mostraram que a incidência de MACE total foi significativamente maior em pacientes com escores de risco GRACE elevados do que em pacientes com escores de risco GRACE baixos em pacientes com IAMSSST-MINOCA. Eles indicaram que o escore de risco GRACE fornece informações prognósticas potencialmente valiosas sobre resultados clínicos quando aplicado a pacientes MINOCA com IAMSSST.^25^

O escore PRECISE-DAPT pode ser calculado de forma rápida e fácil e fornece uma classificação de risco rápida sem custos adicionais. No entanto, o escore PRECİSE-DAPT, comumente usado para prever o risco de sangramento em pacientes tratados com DAPT após ICP, não foi projetado para previsões de mortalidade em longo prazo. No entanto, alguns estudos demonstraram forte correlação entre o escore PRECISE-DAPT e eventos cardiovasculares. Long et al. descobriram que o escore PRECISE-DAPT está independentemente ligado à extensão da estenose coronariana em pacientes com SCA.^26^ Além disso, em pacientes com IAMCSST, o escore PRECISE-DAPT é um preditor independente de IAMCSST. mortalidade hospitalar após ICP primária.^27^

No presente estudo, descobrimos que um escore PRECISE-DAPT mais alto e níveis mais baixos de hemoglobina estavam associados a um risco aumentado de MACE intra-hospitalar após ajuste para outros fatores. Bassand et al. indicaram que um nível basal baixo de hemoglobina é um preditor independente do risco de sangramento maior na SCA, bem como do risco de morte e IAM.^28^ Esse achado é semelhante ao resultado que encontramos no presente estudo.

Descobriu-se que um PRECISE-DAPT mais alto e uma FEVE mais baixa foram preditores independentes de MACE de acompanhamento. Sabemos muito bem que a FEVE baixa é o indicador prognóstico a longo prazo mais importante na SCA e o nosso achado neste estudo é consistente com esta conclusão.

Entretanto, vale ressaltar que a *odds ratio* para o PRECISE-DAPT se aproxima de 1; isto indica que, apesar da significância estatística, não há diferença marginal no risco. Este achado levanta questões sobre o significado fisiológico desta relação. Esta discrepância entre a significância estatística e o tamanho modesto do efeito, refletido pelo *odds ratio* próximo de 1, sublinha a necessidade de uma interpretação cautelosa dos resultados. Sugere que, embora estatisticamente significativas, as implicações clínicas do escore PRECISE-DAPT na previsão de MACE hospitalares podem não se manifestar como diferenças substanciais no risco entre os pacientes.

Este estudo tem várias limitações. Em primeiro lugar, é um estudo retrospectivo e observacional. Em segundo lugar, foi realizado num único centro terciário. Em terceiro lugar, embora tenham sido incluídos pacientes consecutivos, pode haver um viés de seleção. Como tal, os resultados e a duração dos eventos devem ser interpretados com cautela à luz destas alterações.

## Conclusões

Em pacientes MINOCA com SCA, um escore PRECISE-DAPT elevado foi associado a maiores MACE intra-hospitalares e de longo prazo. Como resultado, o escore PRECISE-DAPT pode ser útil não apenas para identificar um risco elevado de hemorragia, mas também para prever um mau resultado em termos de MACE a longo prazo.

### Destaques

– O escore PRECISE-DAPT prevê o risco de MACE em pacientes MINOCA.
– O nível de hemoglobina e o escore PRECISE-DAPT predizem MACE intra-hospitalar.
– A fração de ejeção e o escore PRECISE-DAPT predizem MACE em longo prazo.
