# Supplementary material for: Valor Prognóstico da Escore PRECİSE DAPT em Resultados de Curto e Longo Prazo em Pacientes MINOCA com Síndrome Coronariana Aguda
Source: Arq Bras Cardiol. 2024 May 7;121(5):e20230791. [Article in Portuguese] doi: 10.36660/abc.20230791 (PMC11185829; doi:10.36660/abc.20230791)
Supplement: Translation

# Translation: Prognostic Value of PRECİSE DAPT Score on Short- and Long-Term Outcomes in MINOCA Patients with Acute Coronary Syndrome

**DOI:** 10.36660/abc.20230791i

**Authors:** Tolga Onuk, Fuat Polat, Bariş Yaylak, Ali Nazmi Çalik, Semih Eren, Şükrü Akyüz

**Affiliations:** 1 Istanbul Turkey Dr. Siyami Ersek Thoracic and Cardiovascular Surgery Education Research Hospital, Istanbul – Turkey; 2 Istanbul Turkey Okan University Faculty of Health Science, Istanbul – Turkey

**Keywords:** MINOCA, Atrial Fibrillation, Myocardial Infarction, Dual Anti-Platelet Therapy

## Abstract

**Background:**

Myocardial infarction with non-obstructive coronary arteries (MINOCA) constitutes a significant subset of acute myocardial infarctions (AMI) with uncertain prognostic markers. Early risk assessment is crucial to identify MINOCA patients at risk of adverse outcomes.

**Objectives:**

This study aimed to evaluate the predictive capacity of the PRECISE-DAPT score in assessing short- and long-term prognoses in MINOCA patients presenting with ST-segment elevation myocardial infarction (STEMI) or non-ST-elevation myocardial infarction (NSTEMI).

**Methods:**

Among 741 MINOCA patients, the PRECISE-DAPT score was computed to analyze its association with in-hospital and follow-up major adverse cardiovascular events (MACE). Parameters showing significance in MACE (+) groups underwent statistical analysis: univariate logistic regression for in-hospital events and univariate Cox regression for follow-up events. For statistical significance, a predefined level of α = 0.05 was adopted. Parameters demonstrating significance proceeded to multiple logistic regression for in-hospital events and multivariate Cox regression for follow-up events.

**Results:**

In-hospital MACE occurred in 4.1% of patients, while 58% experienced follow-up MACE. Hemoglobin levels and the PRECISE-DAPT Score were identified as independent parameters for in-hospital MACE. Furthermore, ejection fraction (EF%) and the PRECISE-DAPT Score emerged as independent predictors of follow-up MACE.

**Conclusions:**

The study revealed that a higher PRECISE-DAPT score was significantly associated with increased risks of both in-hospital and long-term major adverse cardiovascular events in MINOCA patients presenting with acute coronary syndrome (ACS), underscoring the score's potential in risk stratification for this patient cohort.

## Introduction

Patients with myocardial infarction with non-obstructive coronary arteries (MINOCA) account for 6% to 14% of patients of all those with acute myocardial infarction (AMI).^1 - 4^

The diagnosis of MINOCA is dependent on the presence of clinical acute MI and the absence of obstructive coronary artery disease (CAD). Actually, MINOCA is initially considered a valid diagnosis during angiography until other possible causes of troponin elevation are excluded. The underlying causes may be coronary conditions or noncoronary conditions including cardiac or noncardiac disorders.^5^MINOCA can be presented as ST-segment elevation myocardial infarction (STEMI) or non-ST-elevation myocardial infarction (NSTEMI) on an electrocardiogram (ECG), however, NSTEMI myocardial infarction is more common in the MINOCA population.^4 , 6 , 7^MINOCA patients show a better prognosis compared to acute coronary syndrome (ACS) patients with obstructive CAD.^8 - 11^However, MINOCA patients have a worse life expectancy compared to age and sex-matched healthy individuals.^8 - 11^

It has been previously shown that risk scores such as the Grace Risk Score are associated with the prognosis in the MINOCA population.^12^The PRECISE-DAPT score, which predicts bleeding risk in patients undergoing stent implantation and subsequent dual antiplatelet therapy (DAPT), was developed to predict bleeding risk in patients treated with DAPT after percutaneous coronary intervention (PCI). This score is calculated by five items (age, white blood cell count, hemoglobin level, creatinine clearance, and history of spontaneous bleeding) and patients with a score of > 25 are at a high risk of bleeding.^13^The current guidelines recommend using the PRECISE-DAPT score for bleeding risk stratification, and a score of > 25 indicates that the DAPT period should be shorter than 3 to 6 months in patients with a score of < 25.^14 - 16^In a recent study, a high PRECISE-DAPT score was associated with higher long-term all-cause mortality in patients with AMI.^17^Thus, the present study aims to assess the performance of the PRECISE-DAPT score in predicting the short and long-term prognosis in MINOCA patients presenting with acute coronary syndrome.

## Methods

The study is retrospective and observational. We included 7300 patients admitted to our hospital with the diagnosis of STEMI or NSTEMI between April 2013 and December 2022. Among the 7300 patients who underwent percutaneous intervention, a subset of 741 individuals received a diagnosis of acute coronary syndrome and MINOCA. Coronary angiography was performed in all patients. Patients who did not have coronary stenosis of 50% or more in any coronary artery on coronary angiography and who were not diagnosed with spontaneous coronary artery dissection, myocarditis, and Takotsubo cardiomyopathy were classified as MINOCA. In our study, patients with obstructive coronary artery stenosis other than atherosclerotic coronary artery diseases were not included in the study and were not classified as MINOCA. The flowchart of the study population is shown in Figure 1 .

**Figure 1:** The flowchart of the study population.

The PRECISE-DAPT score, age, creatinine clearance, white blood cell count, hemoglobin, and previous bleeding history were calculated for each case using a web calculator ( http://www.precisedaptscore.com ). Clinical, biochemical, interventional, ECG and transthoracic echocardiography data were obtained from the hospital's electronic data set. Echocardiography was performed to determine left ventricular ejection fraction (LVEF). The proportion of patients with a previous history of heart attack or coronary revascularization was specified and included in the study. All patients, including those with a previous heart attack or history of revascularization, underwent emergency PCI. Medications, including the initiation and duration of dual antiplatelet therapy and anticoagulation therapy, were prescribed at the discretion of the attending physician upon discharge. The major in-hospital cardiovascular events were identified as cardiovascular mortality, stroke, and major bleeding. For assessing long-term major cardiovascular events, the following were considered: cardiac vascular death, non-cardiovascular death, stroke, major bleeding, percutaneous intervention, and myocardial infarction. The in-hospital MACE of all patients was obtained from the patient's medical records. The long-term major cardiovascular events of all patients were obtained from the patient's medical records, the relevant doctors at the hospital to which the patients were referred, or by telephoning the patients. The study design was approved by the local ethical committee and was conducted following the Helsinki Declaration.

### Statistical analysis

The data analysis utilized SPSS software (Version 23.0, SPSS, Inc., Chicago, IL). Continuous variables were assessed for normality, employing visual inspection through histograms, probability plots, and the Kolmogorov-Smirnov test. Levene's test was utilized to assess homogeneity. Continuous variables were presented as mean ± standard deviation for normally distributed data and as median with interquartile range for non-normally distributed data.

Additionally, statistical tests including unpaired Student's t-test and Mann-Whitney test were applied to assess differences in continuous variables between groups, ensuring a comprehensive analysis of continuous variable associations. While categorical variables were given as absolute and relative frequencies, differences in categorical variables between groups were assessed using the Chi-square or Fisher's exact test.

In the in-hospital analysis, parameters demonstrating a statistically significant difference in the MACE (+) group underwent initial evaluation through univariate logistic regression analysis, followed by multiple logistic regression analysis for significant results. For the follow-up analysis, parameters exhibiting a statistically significant difference in the MACE (+) group were initially examined using univariate Cox regression analysis, followed by multivariate Cox regression for significant findings.

Subsequently, factors independently predicting MACE underwent ROC analysis to determine sensitivity and specificity. The cut-off value for the PRECISE-DAPT score, determined by the highest sensitivity and specificity, was utilized for Kaplan-Meier analysis to compare long-term survival. The level of significance adopted in the statistical analysis was 5%. Additionally, analyses were conducted ensuring basic assumptions for logistic regression including independence of errors, linearity in the logit for continuous variables, absence of multicollinearity, and lack of strongly influential outliers.

## Result

A total of 741 patients were included in the study. Long-term follow-up data of 734 patients were obtained. The median follow-up period of the total of 734 patients included in the study was 56.8 (Interquartile range [IQR] 38.3-76.5) months. In-hospital MACE occurred in 4.1% of the patients and follow-up MACE occurred in 58% of the patients ( Figure 2 ).

**Figure 2:** In-hospital and Follow-up MACE of the patients.

The average age of the patients was 61.4±14.1 years, and the male gender was predominant (58.5%). The mean PRECISE DAPT Score of the patients was 23.6 ± 14.4. Almost all patients (98.8%) received ASA 300 mg before or during the procedure. The majority of patients received Clopidogrel 300 mg before or during the procedure. The proportion of patients receiving Ticagrelor at baseline was 11.7%. Other characteristic features of all patients are shown in Table 1 .

**Table 1:** Clinical and demographic characteristics of patients

Patient characteristics	n = 741
Sex (male) (%)	434(58.5)
Age (years) (mean±SD)	61.4±14.1
BMI (kg/m²) (mean±SD)	28.21±5.35
Hypertension (%)	369(49.7)
Diabetes Mellitus (%)	224(30.2)
Smoking (Active) (%)	305(41.1)
Dyslipidemia (%)	39(5.3)
COPD (%)	29(3.9)
Cardiogenic shock on admission (%)	40(5.4)
Killip III-IV on admission (%)	42(5.7)
LBBB on ECG (%)	68(9.2)
Prior heart attack (%)	49(6.6)
prior revascularization (%)	71(9.6)
Prior cerebrovascular accident (%)	29(3.9)
PRECISE DAPT Score (mean±SD)	23.6±14.4
Prior warfarin use (%)	42(5.7)
Prior NOAC use (%)	29(3.9)
WBC (10^3^/uL) (mean±SD)	10.3±4.1
Creatinine (mg/dL) (median, IQR)	1.0(0.8-1.3)
Hemoglobin (g/dL) (mean±SD)	12.5±2.1
LVEF% (mean±SD)	46±12.7
Ticagrelor (%)	87(11.7)
Clopidogrel 300 mg (%)	638(86)
Clopidogrel 600 mg (%)	8(1.1)
ASA (%)	733(98.8)
Beta-blocker (%)	693(93.4)
Calcium channel blocker (%)	172(23.2)
ACEi/ARB (%)	574(77.4)
PPI (%)	734(99.1)
Statin (%)	618(83.3)
LMWH before the procedure (%)	667(90.6)

ACEi/ARB: Angiotensin-converting enzyme inhibitors/Angiotensin II receptor blockers; ASA: Acetylsalicylic acid; BMI: Body mass index; COPD: Chronic obstructive pulmonary disease; ECG: Electrocardiography; IQR: Interquartile range; LBBB: Left bundle branch block; LMWH: Low molecular weight heparin; LVEF: Left ventricle ejection fraction; NOAC: Non-Vitamin K antagonist oral anticoagulants; PPI: Proton pump inhibitor; SD: Standard deviation; WBC: White blood cell.

The patients were divided into two groups with (+) in-hospital MACE and without (-) in-hospital MACE ( Table 2 ). In the MACE (+) group there were higher rates of cardiogenic shock (p=0.008), higher mean BMI (p=0.025), and active smokers. The PRECISE DAPT score was two times higher (22.6% vs 43.9%) in MACE (+) patients than in MACE (-) patients. In addition, MACE (+) patients had lower rates of ACE inhibitors/ARBs and statin prescriptions, lower hemoglobin levels, lower LVEF%, and higher creatinine levels.

**Table 2:** Clinical and demographic characteristics of patients with or without in-hospital MACE groups

Patient characteristics	MACE (-) in Hospital (n=709)	MACE (+)in Hospital (n=32)	p value
Sex (male) (%)	414(58.4)	19(59.4)	0.533
Age (years) (mean±SD)	61.6±14	57.1±14.3	0.071
BMI (kg/m²) (mean±SD)	28.09±5.02	30.05±6.86	0.008 *
Hypertension (%)	352(49.6)	17(53.1)	0.419
Diabetes Mellitus (%)	215(30.3)	9(28.1)	0.482
Smoking (Active) (%)	285(40.2)	19(59.4)	0.025 *
Dyslipidemia (%)	38(5.4)	1(3.2)	0.488
COPD (%)	28(3.9)	1(3.1)	0.640
Cardiogenic shock on admission (%)	35(4.9)	5(15.6)	0.024 *
Killip III-IV on admission (%)	37(5.2)	4(12.5)	0.094
LBBB on ECG (%)	64(9)	4(12.5)	0.337
Prior heart attack (%)	46(6.5)	3(9.4)	0.127
prior revascularization (%)	66 (9.3)	5(15.6)	0.089
Prior cerebrovascular accident (%)	28(3.9)	1(3.2)	0.640
PRECISE DAPT Score (mean±SD)	22.6±13.8	43.9±13.9	<0.001 *
Prior warfarin use (%)	41(5.8)	1(3.1)	0.444
Prior NOAC use (%)	28(4)	1(3.1)	0.640
WBC (10^3^/uL) (mean±SD)	10.2±4.1	11.5±4.1	0.053
Creatinine (mg/dL) (median, IQR)	0.9 (0.7-1.2)	1.3(0.9-1.6)	0.003 *
Hemoglobin (g/dL) (mean±SD)	12.6±1.9	9.4±1.7	<0.001 *
LVEF% (mean±SD)	46.3±12.4	38.2±15.5	<0.001 *
Ticagrelor (%)	83(11.7)	4(12.5)	0.531
Clopidogrel 300 mg (%)	609(85.9)	28(87.5)	0.525
Clopidogrel 600 mg (%)	8(1.1)	0(0)	0.701
ASA (%)	701(98.9)	31(96.99	0.329
Beta-blocker (%)	665(93.8)	28(87.5)	0.146
Calcium channel blocker (%)	161(22.7)	11(34.4)	0.098
ACEi/ARB (%)	562(79.3)	12(37.5)	0.001 *
PPI (%)	702(99)	32(100)	0.733
Statin (%)	597(84.2)	21(65.6)	0.011 *
LMWH before the procedure (%)	638(90.6)	29(90.6)	0.593

ACEi/ARB: Angiotensin-converting enzyme inhibitors/Angiotensin II receptor blockers; ASA: Acetylsalicylic acid; BMI: Body mass index; COPD: Chronic obstructive pulmonary disease; ECG: Electrocardiography; IQR: Interquartile range; LBBB: Left bundle branch block; LMWH: Low molecular weight heparin; LVEF: Left ventricle ejection fraction; MACE: Major adverse cardiovascular events; NOAC: Non-Vitamin K antagonist oral anticoagulants; PPI: Proton pump inhibitor; SD: Standard deviation; WBC: White blood cell.

* Significant p value.

When statistically significant parameters between the two groups were evaluated by univariate logistic regression analysis, active smoking, shock on admission, PRECISE DAPT Score, creatinine level, hemoglobin level, LVEF%, ACEi/ARB use, statin use, and BMI were the factors retained significantly. Applying these parameters to multiple logistic regression analyses, hemoglobin level, and PRECISE DAPT Score were found to be independent parameters for in-hospital MACE ( Table 3 ).

**Table 3:** Univariate and multiple logistic regression analysis results of groups with and without MACE during the in-hospital

Patient characteristics	Univariate Analysis			Multiple Logistic Regression Analysis		
	OR	95%CI	p	OR	95%CI	p
Smoking (Active)	2.174	1.051-4.473	0.035			
Shock on admission	3.566	1.299-9.820	0.014			
PRECISE DAPT Score	1.100	1.071-1.131	<0.001	1.039	1.003-1.077	0.034
Creatinine	1.304	1.075-1.581	0.007			
Hemoglobin	0,405	0,316-0,520	<0.001	0,482	0,356-0,652	<0.001
LVEF	0,955	0,930-0,981	0.001			
ACEi/ARB	0,157	0,075-0,328	<0.001			
Statin	0,358	0,168-0,763	0.008			
BMI	1.073	0,030-5,522	0.019			

IACEi/ARB: Angiotensin-converting enzyme inhibitors/Angiotensin II receptor blockers; BMI: Body mass index; LVEF: Left ventricle ejection fraction.

The follow-up patients were divided into two groups with (+) follow-up MACE and without (-) follow-up MACE ( Table 4 ). MACE was more frequent in patients with cardiogenic shock, Killip III-IV at presentation, and in patients with LBBB on ECG. The PRECISE DAPT score was more frequent (18.1% vs 27.1%) in MACE (+) patients than in MACE (-) patients. MACE (+) patients had higher rates of warfarin and CCB use and lower rates of clopidogrel and ticagrelor use longer than 1 year, ACEİ/ARB, and PPI use. In addition, lower hemoglobin and LVEF% and higher creatinine and WBC levels were observed in MACE (+) patients.

**Table 4:** Clinical and demographic characteristics of patients with or without follow-up MACE groups

Patient characteristics	MACE (-) Follow-up (n=308)	MACE (+) Follow-up (n=426)	p value
Sex (male) (%)	172(55.8)	258(60.6)	0.114
Age (years) (mean±SD)	61.7±13.2	61.1±14.5	0.563
BMI (kg/m²) (mean±SD)	28.7±5.2	27.8±4.9	0.113
Hypertension (%)	156(50.6)	208(48.8)	0.340
Diabetes Mellitus (%)	93(30.2)	131(30.8)	0.469
Smoking (Active) (%)	123(39.9)	178(41.8)	0.335
Dyslipidemia (%)	20(6.5)	19(4.5)	0.248
COPD (%)	12(3.9)	17(4.0)	0.554
Cardiogenic shock on admission (%)	9(2.9)	28(6.6)	0.018 *
Killip III-IV on admission (%)	6(1.9)	32(7.5)	<0.001 *
LBBB on ECG (%)	15(4.9)	52(12.2)	<0.001 *
Prior heart attack (%)	20(6.5)	29(6.8)	0.405
Prior revascularization (%)	29(9.4)	42(9.9)	0.234
Prior cerebrovascular accident (%)	12(3.9)	17(4.0)	0.554
PRECISE DAPT Score (mean±SD)	18.1±13.2	27.1±13.6	<0.001 *
Prior warfarin use (%)	11(3.6)	31(7.3)	0.023 *
Prior NOAC use (%)	11(3.6)	18(4.2)	0.406
WBC (103/uL) (mean±SD)	9.5±3.8	10.4±4.3	0.008 *
Creatinine (mg/dL) (median, IQR)	1.0(0.8-1.3)	1.3(1.0-1.6)	<0.001 *
Hemoglobin (g/dL) (mean±SD)	12.9±2.1	12.1±2.1	<0.001 *
LVEF% (mean±SD)	51.5±10.6	42.2±12.4	0.001 *
ASA (%)	305(99)	420(98.6)	0.433
Beta-blocker (%)	291(94.5)	397(93.2)	0.291
Calcium channel blocker (%)	52(16.9)	120(28.2)	<0.001 *
ACEi/ARB (%)	268(87)	306(71.9)	<0.001 *
PPI (%)	283(91.9)	333(78.2)	<0.001 *
P2Y12 inhibitor >1 year (%)	214(69.5)	217(50.9)	<0.001 *

IECA/BRA: Inibidores da enzima conversora de angiotensina/bloqueadores dos receptores da angiotensina II; AAS: Ácido acetilsalicílico; IMC: Índice de massa corporal; DPOC: Doença pulmonar obstrutiva crônica; ECG: Eletrocardiografia; IQR: Intervalo interquartil; BRE: Bloqueio de ramo esquerdo; HBPM: Heparina de baixo peso molecular; FEVE: Fração de ejeção do ventrículo esquerdo; MACE: Principais eventos cardiovasculares adversos; NOAC: Anticoagulantes orais não antagonistas da vitamina K; IBP: Inibidor da bomba de prótons; DP: Desvio padrão.

* Valor p significativo.

When statistically significant parameters between the two groups were evaluated by univariate Cox regression analysis, LBBB on ECG, PRECISE DAPT Score, WBC level, creatinine level, hemoglobin level, LVEF, clopidogrel and ticagrelor use longer than 1 year, ACEi/ARB and statin use were the factors retained significantly. Applying these parameters to a multivariate Cox regression analysis (enter method), LVEF% and PRECISE DAPT Score were found to be the independent predictors of follow-up MACE ( Table 5 ).

**Table 5:** Univariate and multivariate Cox regression analysis results of groups with and without MACE during follow-up

Patient characteristics	Univariate Analysis			Multivariate Analysis		
	HR	95%CI	p value	HR	95%CI	p value
Shock on admission	1.383	0.942-2.030	0.097			
Killip III-IV on admission	1.432	0.998-2.054	0.051			
LBBB on ECG	1.381	1.024-1.862	0.034 *			
PRECISE DAPT Score	1.019	1.013-1.025	<0.001 *	1.013	1.006-1.020	<0.001
Prior warfarin use	1.096	0.760-1.581	0.624			
WBC	1.027	1.006-1.048	0.012 *			
Creatinine	1.104	1.025-1.190	0.009 *			
Hemoglobin	0.902	0.862-0.945	0.001 *			
LVEF	0.975	0.968-0.982	<0.001 *	0.980	0.972-0.987	<0.001
CCB	1.319	1.067-1.630	0.010 *			
ACE/ARB	0.710	0.574-0.878	0.002 *			
Statin	0.698	0.554-0.879	0.002 *			
P2Y12 inhibitor >1 year	0.799	0.660-0.968	0.022 *			

ACEi/ARB: Angiotensin-converting enzyme inhibitors/Angiotensin II receptor blockers; ECG: Electrocardiography; LBBB: Left bundle branch block; LVEF: Left ventricle ejection fraction; WBC: White blood cell.

* Significant p value.

We evaluated the sensitivity and specificity of the independent predictors of MACE using ROC analysis ( Figure 3 ). Since the AUC area was low in the ROC analysis performed according to LVEF, sensitivity and specificity calculations were not made. The AUC level of the PRECISE DAPT score was sufficiently high. When the PRECISE DAPT score was > 20.5, sensitivity was determined as 72%, specificity as 62%, positive predictive value as 65.5%, and negative predictive value as 68.9%.

**Figure 3:** ROC analysis of PRECISE DAPT Score between groups in multivariate Cox proportional regression analysis.

We used the 20.5% cut-off value to evaluate long-term survival by Kaplan-Meier analysis ( Figure 4 ), which resulted in a Chi-square of 43.29, long-rank p<0.001. The MACE rate between groups was observed to differ in the first follow-up year.

**Figure 4:** Long-term survival according to PRECISE DAPT score by Kaplan-Meier analysis.

## Discussion

In this study, we found that a high PRECISE-DAPT score is a significant independent predictor of in-hospital and long-term MACE in MINOCA patients with acute coronary syndrome. To the best of our knowledge, this is the first study to report that in-hospital and long-term MACE is significantly higher in MINOCA patients with acute coronary syndrome with a high PRECISE-DAPT score. ROC analysis demonstrated that the PRECISE-DAPT score had a moderate discrimination ability to stratify ACS-MINOCA patients by their risk of MACE. The data showed that the PRECISE-DAPT risk score has prognostic value in patients with ACS-MINOCA ( Central Illustration ).

Numerous studies have recently been published on the prognostic value of MINOCA. In a recent review by Pasupathy et al., MINOCA had a lower all-cause mortality rate than those with MI-CAD.^4^In a meta-analysis published by Pizzi et al., all cardiovascular outcome event rates (MACE, all deaths, cardiac death, MI, and all deaths plus MI) in non-obstructive CAD were significantly lower than those in obstructive CAD.^18^A recent study on Chinese MINOCA patients revealed that MACE (cardiovascular deaths, non-fatal MI, strokes, heart failures, and cardiovascular-related rehospitalizations) was lower in the MINOCA group than in the MI-CAD group at the 1-year follow-up.^19^In some studies, it has been shown that the prognosis of MINOCA is better than MI-CAD patients.^4 , 7 , 20^Some studies indicated that patients with MINOCA had clinical outcomes that were similar to those of MI-CAD patients.^21^Dreyer et al., indicated a worse prognosis at 1-year follow-up after AMI in elderly MINOCA patients over 65 years of age compared to those with MI-CAD patients.^22^In addition to all these studies, 2 studies found higher MACE incidence in MINOCA patients compared to the normal population.^23 , 24^

Risk assessment tools can help to predict the probability of mortality and morbidity. To our knowledge, there is currently only one risk score for predicting the prognosis of MINOCA. Yin, Guoqing, et al., showed that the incidence of total MACE was significantly higher in patients with high GRACE risk scores than in patients with low GRACE risk scores in NSTEMI-MINOCA patients. They indicated that the GRACE risk score provides potentially valuable prognostic information on clinical outcomes when applied to MINOCA patients with NSTEMI.^25^

The PRECISE-DAPT score can be quickly and easily calculated and provides a rapid risk classification without additional costs. However, the PRECİSE-DAPT score which is commonly used to predict bleeding risk in patients treated with DAPT after PCI, is not designed for long-term mortality predictions. Nevertheless, some studies have demonstrated a strong correlation between the PRECISE-DAPT score and cardiovascular events. Long et al. found that the PRECISE-DAPT score is independently linked to the extent of coronary stenosis in patients with acute coronary syndrome.^26^Moreover, in patients with STEMI, the PRECISE-DAPT score is an independent predictor of in-hospital mortality after primary PCI.^27^

In the current study, we found that a higher PRECISE-DAPT score and lower hemoglobin levels were all associated with an increased risk of in-hospital MACE after adjusting for other factors. Bassand et al. indicated that a low baseline hemoglobin level is an independent predictor of the risk of major bleeding in ACS as well as of the risk of death and MI.^28^This finding is similar to the result we found in the current study.

Higher PRECISE-DAPT and lower LVEF were found to be the independent predictors of follow-up MACE. We know very well that low LVEF is the most important long-term prognostic indicator in ACS and our finding in this study is consistent with this conclusion.

However, it is noteworthy that the odds ratio for PRECISE-DAPT approaches 1; This indicates that despite statistical significance, there is no marginal difference in risk. This finding raises questions regarding the physiological significance of this relationship. This discrepancy between statistical significance and the modest effect size, as reflected by the odds ratio close to 1, underscores the need for cautious interpretation of the findings. It suggests that while statistically significant, the clinical implications of the PRECISE-DAPT score in predicting in-hospital MACE may not manifest as substantial differences in risk among patients.

This study has several limitations. Firstly, it is a retrospective and observational study. Secondly, it was conducted at a single tertiary center. Thirdly, while consecutive patients were enrolled, there may be a selection bias. As such, the results and duration of events should be interpreted with caution in light of these changes.

## Conclusions

In MINOCA patients with ACS, a high PRECISE-DAPT score was associated with higher in-hospital and long-term MACE. As a result, the PRECISE-DAPT score can be useful not just for identifying a high risk of bleeding, but also for forecasting a poor outcome in terms of long-term MACE.

### Highlights

– PRECISE-DAPT score predicts MACE risk in MINOCA patients.
– Hemoglobin level and PRECISE-DAPT score predict in-hospital MACE.
– Ejection fraction and PRECISE-DAPT score predict long-term MACE.
